# A combined transcriptome - miRNAome approach revealed that a *kinesin* gene is differentially targeted by a novel miRNA in an apomictic genotype of *Eragrostis curvula*

**DOI:** 10.3389/fpls.2022.1012682

**Published:** 2022-09-30

**Authors:** María Cielo Pasten, José Carballo, Jimena Gallardo, Diego Zappacosta, Juan Pablo Selva, Juan Manuel Rodrigo, Viviana Echenique, Ingrid Garbus

**Affiliations:** ^1^ Centro de Recursos Naturales Renovables de la Zona Semiárida (CERZOS), Universidad Nacional del Sur-Consejo Nacional de Investigaciones Científicas y Técnicas (CONICET), Bahía Blanca, Argentina; ^2^ Departamento de Agronomía, Universidad Nacional del Sur (UNS), Bahía Blanca, Argentina; ^3^ Departamento de Biología, Bioquímica y Farmacia, Universidad Nacional del Sur (UNS), Bahía Blanca, Argentina

**Keywords:** *Eragrostis curvula*, apomixis, sRNA libraries, miRNA, miRNA-mRNA interaction

## Abstract

Weeping lovegrass (*Eragrostis curvula* [Shrad.] Nees) is a perennial grass typically established in semi-arid regions, with good adaptability to dry conditions and sandy soils. This polymorphic complex includes both sexual and apomictic cytotypes, with different ploidy levels (2x-8x). Diploids are known to be sexual, while most polyploids are facultative apomicts, and full apomicts have also been reported. Plant breeding studies throughout the years have focused on achieving the introgression of apomixis into species of agricultural relevance, but, given the complexity of the trait, a deeper understanding of the molecular basis of regulatory mechanisms of apomixis is still required. Apomixis is thought to be associated with silencing or disruption of the sexual pathway, and studies have shown it is influenced by epigenetic mechanisms. In a previous study, we explored the role of miRNA-mRNA interactions using two contrasting *E. curvula* phenotypes. Here, the sexual OTA-S, the facultative Don Walter and the obligate apomictic Tanganyika cDNA and sRNA libraries were inquired, searching for miRNA discovery and miRNA expression regulation of genes related to the reproductive mode. This allowed for the characterization of seven miRNAs and the validation of their miRNA-target interactions. Interestingly, a *kinesin* gene was found to be repressed in the apomictic cultivar Tanganyika, targeted by a novel miRNA that was found to be overexpressed in this genotype, suggestive of an involvement in the reproductive mode expression. Our work provided additional evidence of the contribution of the epigenetic regulation of the apomictic pathway.

## Introduction

Apomixis can be described as asexual reproduction through seeds, and it comprises three central stages: apomeiosis (absence of meiosis), parthenogenesis (absence of fertilization) and autonomous endosperm development or pseudogamy (Nogler, 1984; Schmidt, 2020). It has been observed that apomixis is inherited as a dominant trait, and that the three developmental stages are controlled by independent loci in several species (Grossniklaus, 2001; Vijverberg and van Dijk, 2007).

There is a wide variety of apomictic mechanisms, which can be explained by the fact that apomixis emerged independently multiple times during evolution. It is theorized that apomixis arises from spatial and temporal changes in the expression of genes involved in sexual reproduction. The regulation guided by the availability of specific expression of the genes PAR1 and PsASGR-BabyBoom-Like has been observed for parthenogenesis in Pennisetum and Taraxacum (Underwood et al., 2022, Khanday et al., 2019). However, these genes *per se* are insufficient to transfer apomixis to economically important crops, since no genes linked to apomeiosis were found in apomictic species.

It has been found that the reproductive state can be influenced by environmental cues (Rodrigo et al., 2017; Gao, 2018; Schmidt, 2020). In numerous species, differences in photoperiod, salt stress and heat stress result in similar responses, adding to the now accepted notion that reproduction adjustments can take place, and that they appear to have a significant role in the adaptation of facultative apomicts (Gounaris et al., 1991; Hussey et al., 1991; Carman et al., 2015; Klatt et al., 2016).

There is evidence that shows that DNA methylation, chromatin modifications and noncoding RNAs have a central role in the regulation of gene expression at transcriptional and post-transcriptional levels (Pikaard and Scheid, 2014; Wei et al., 2017; Higo et al., 2020; Brukhin and Albertini, 2021; Carballo et al., 2021). The study of epigenetics in the last years has established that these mechanisms are key to the understanding of numerous processes in plants. Among the noncoding RNAs, the most abundant and important group are the small RNAs (sRNAs), that target coding or non-coding RNAs for silencing, *via* guided degradation or translational repression (Borges and Martienssen, 2015; Nejat and Mantri, 2018). MicroRNAs (miRNAs) are a large class of sRNAs derived from DICER-LIKE (DCL) processing of single-stranded precursors, known to participate mostly in post-transcriptional gene silencing (PTGS) (Borges and Martienssen, 2015). miRNAs regulate gene expression by specifically targeting messenger RNAs (mRNA) in association with the RNA-induced silencing complex (RISC), consisting mainly of ARGONAUTE (AGO) proteins, mainly AGO1, AGO4 and AGO10 (Neumeier and Meister, 2021) and acting either *via* direct degradation or *via* inhibition of translation, pairing with the 3’ UTR region of the target (Melo and Melo, 2013).

miRNAs have central regulatory roles and affect processes such as reproduction, fertility, and leaf and flower morphogenesis, among others (Aukerman and Sakai, 2003; Palatnik et al., 2003; Schwab et al., 2005; Li et al., 2015; Ripoll et al., 2015). In *A. thaliana, Z. bungeanum* and some species of the genus Boechera, specific miRNAs have been identified (miR156/157, miR167 y miR172c) related to functions such as flower organogenesis, reproductive mode, and male and female fertility (Xing et al., 2013; Ru et al., 2006; Amiteye et al., 2011; Fei et al., 2021). Differential expression analyses of miRNAs in regards to ploidy and reproductive mode have also taken place (Amiteye et al., 2013; Ortiz et al., 2019).

*Eragrostis curvula* [Shrad.] Nees (weeping lovegrass), is a perennial grass belonging to the Poaceae family, native of Southern Africa, and it is commonly found in semi-arid regions around the world due to its ability to adapt to drought conditions and low fertility soils (Covas, 1991). This species constitutes a morphologically and evolutively diverse group, and it includes cytotypes with different ploidy levels (2x to 8x), which can reproduce sexually or by diplosporous apomixis (obligate or facultative) (Leigh, 1960; Voigt and Burson, 1983).

Due to its specific apomictic development, *E. curvula* has become one of several models for the study of apomictic mechanisms and was widely studied over the last years, obtaining information about its genome (Carballo et al., 2019), cytoembriology (Zappacosta et al., 2014), the position of the apomixis locus (Zappacosta et al., 2019), expression of candidate genes (Cervigni et al. 2008a, and Cervigni et al. 2008b; Garbus et al., 2017; Selva et al., 2017; Selva et al., 2020). More recently, the methylation status of facultative, fully sexual and fully apomictic genotypes was assessed, finding that facultative genotypes have a higher number of methylated positions (Carballo et al., 2021). Even when the methylation status could be interpreted as a consequence of the different reproduction systems rather than the cause (Mau et al., 2022), the tetraploid nature and the independent genetic origin of the three genotypes analyzed in the mentioned study is supporting the hypothesis that links the methylation status with the reproductive mode. This behavior shows the importance of epigenetic mechanisms in more dynamic reproductive conditions.

The scarce diploid plants of *E. curvula* (2n = 2x = 20) reproduce sexually, while polyploids mostly undergo facultative pseudogamous diplosporous apomixis (Leigh, 1960; Voigt and Burson, 1983). In this process, a megasporocyte avoids meiosis and experiences two rounds of mitotic division, generating four non-reduced nuclei at maturity: two synergid cells, an egg cell and one polar nucleus (Crane, 2001). The development of the non-reduced tetranucleate embryo sac is specific of this grass, that also undergoes parthenogenesis but requires fertilization of the polar nucleus for the development of the endosperm (Grimanelli et al., 2001; Crane, 2001).

In a previous study conducted using two contrasting *E. curvula* phenotypes, the sexual OTA-S and the apomictic Tanganyika, we investigated the role of miRNA-mRNA interactions in apomixis using a transcriptomic library and small RNA libraries (Garbus et al., 2019). Two genes were identified, a MADS-box transcription factor gene and a transposon, that were found to be repressed in the sexual genotype, most likely due to interactions with miRNAs, suggesting that a repressive effect on gene expression exerted by miRNAs might be involved in the expression of apomixis in *E. curvula* (Garbus et al., 2019).

In the current study we expanded the comprehension of the participation of miRNAs in the expression of apomixis in *E. curvula*, through its known function in mRNA degradation, by including the facultative apomictic material Don Walter. In this way, we analyzed the differential expression of miRNAs and their targets focusing on all the possible comparisons among the sexual cultivar OTA-S, the apomictic cultivar Tanganyika and the facultative Don Walter. There were described seven miRNAs, including their mature sequences, the pri-miRNA and pre-miRNA precursors, as well as their specific interaction with their target mRNAs. A specific regulation by genotype for the transcript for a *kinesin* gene that was found, being repressed in the apomictic cultivar Tanganyika, whereas the related miRNA expression was the opposite, i.e., overexpressed in the mentioned genotype. Our results support the previous evidence of the role of miRNAs in transcript regulation in apomixis.

## Materials and methods

### Plant materials

The experiments were carried out using three tetraploid (2n = 4x = 40) *E. curvula* cultivars: the sexual OTA-S (USDA: PI574506), the full apomictic Tanganyika (USDA: PI234217) and the facultative apomictic Don Walter. The plants were grown in the greenhouse in 10 L pots, under natural light conditions (38°43’0″S, 62°16’0″O), at 25˚C +/- 4°C.

### cDNA and sRNA libraries

To conduct the analyses reported in this work, we made use of the cDNA libraries previously constructed by our laboratory for genotypes OTA-S, Tanganyika and Don Walter (Garbus et al., 2017; Selva et al., 2020). OTA-S and Tanganyika cDNA libraries were constructed, sequenced and assembled as reported in Garbus et al. (2017). Briefly, libraries were obtained from spikelets with basal flowers at the beginning of anthesis, containing embryo sacs at all developmental stages, in biological duplicates for each genotype, naming O2P1 and O2P2 those derived from OTA-S, whereas the ones constructed from Tanganyika were called T3P1 and T3P2.

These libraries were deposited in the Sequence Reads Archive (SRA) database at NCBI as BioProject 358210 (Biosamples SAMN06167423 and SAMN06167424), whereas the Transcriptome Shotgun Assembly (TSA) project has been deposited at DDBJ/EMBL/GenBank under the accession GFVM00000000. cDNA libraries from the cultivar Don Walter were made in triplicates from spikelets with basal flowers at the beginning of anthesis, containing embryo sacs at all developmental stages, and were named DW1, DW2 and DW3. The procedures followed for library construction are described in Selva et al. (2020). The TSA Assembly project was deposited at DDBJ/EMBL/GenBank under the accession GIQX00000000.

Concerning small RNAs, libraries from OTA-S and Tanganyika were available (Garbus et al., 2019). They were constructed starting from the small RNA fraction obtained in the separation from the large RNA fraction used for the cDNA libraries (Garbus et al., 2017; Garbus et al., 2019), and thus biological replicates for each genotype lead to the sRNA libraries called O2P1, O2P2, T3P1 and T3P2. The procedure followed for library construction, sequencing and analysis is detailed in Garbus et al. (2019).

On the other hand, sRNA libraries from Don Walter were constructed aiming at this study, as detailed in the next section.

### Small RNA libraries from Don Walter construction, sequencing and processing

Starting from 30 mg of fresh tissue from each sample, ground to a fine powder using liquid nitrogen, the total RNA was extracted as two fractions, small and large RNA, which include RNA sequences smaller and larger than 200 bp, respectively, using a commercial NucleoSpin^®^ miRNA kit (Macherey-Nagel, Düren, Germany) according to the manufacturer’s instructions (Selva et al., 2020). The small RNA fraction was sequenced in biological triplicates, DW1, DW2 and DW3, in 50 bp single end reads through Illumina MiSeq platform in the Polo d’Innovazione di Genomica, Genetica e Biologia (Polo GGB), in Siena, Italy.

After sequencing, the adapters of the raw reads were removed and trimmed using Cutadapt (Martin, 2011) with the following parameters: Phred quality score cutoff 30, minimum read length 18. The reads of the seven samples were collapsed to remove redundancy, using the script collapse_reads.pl. These libraries were deposited in the Sequence Reads Archive (SRA) database at NCBI as BioProject PRJNA855479 (Biosamples SAMN29493390, SAMN29493391 and SAMN29493392).

### miRNA prediction and target identification

The software miRDP2 was used to predict miRNAs, using the Don Walter genome assembly to discover novel and known miRNAs. The first step in miRDP2 was to align reads against the Rfam database in order to identify and discard known rRNA, tRNA, scRNA, snRNA and snoRNAs. miRDP2 reports the mature, pri-miRNA and pre-miRNAs.

The plot of the hairpins was performed using the RNAplot script from ViennaRNA package 2.0 (Lorenz et al., 2011). The predicted mature miRNA file was indexed and each sample was mapped with the software Bowtie (Langmead, 2010) with 0 mismatches to quantify mature miRNAs. A matrix with the reads mapping on the predicted mature miRNA was constructed, and filtered when a mature miRNA was absent in at least 5 samples and the sum of all the mapping reads were less than 10. The R package DESeq2 (Love et al., 2014) was used to perform the normalization and the expression analysis. A principal component analysis was conducted to reduce complexity and compare all the samples. Comparisons were made between the full apomictic cultivar Tanganyika and the sexual cultivar OTA-S, and the facultative cultivar Don Walter, hereafter referred as APOvsSEX and APOvsFAC, respectively. The facultative cultivar Don Walter was further compared to the sexual OTA-S, hereafter referred as FACvsSEX. A mature miRNA was considered to be differentially expressed when the log2FoldChange was >1 and <1 for upregulated and downregulated mature miRNAs, respectively, and when padj<0.05.

Target identification was performed by the software psRNAtarget (Dai et al., 2018) using the genome annotation and the predicted mature miRNAs as input. Finally, in order to correlate the miRNA target with the transcript expression level, a differential expression analysis was conducted using previously sequenced transcriptomic reads from the three genotypes (Garbus et al., 2017; Selva et al., 2020). For these data sets, the software hisat2 (Kim et al., 2019) was used for mapping, and DESeq2 (Love et al., 2014) was used to perform the expression analysis.

### DNA and RNA extraction

DNA and RNA were extracted from the cultivars OTA-S, Tanganyika and Don Walter aiming to validate the *in silico* observations. To carry out this purpose, three plants from each cultivar were grown reproducing the conditions applied for the construction of the libraries. The DNA was extracted from fresh leave tissue in biological triplicates for each cultivar, following a protocol based on cetyltrimethylammonium bromide (CTAB). Briefly, plant material was initially frozen and powdered in liquid nitrogen using a plastic pestle in a 1.5 ml Eppendorf tube. A total of ~50 mg of powdered tissue was incubated at 65°C in preheated CTAB extraction buffer containing 100 mM Tris HCl pH 8, 1.4 M NaCl, 20 mM EDTA pH 8, 2% CTAB (w/v) and 0.5% (v/v) β-mercaptoethanol. Then, chloroform was added to reach the proportion 2:1, collecting the supernatant obtained after a centrifugation step. Finally, DNA was precipitated with one volume of isopropanol, washed with 70% (v/v) ethanol and resuspended in ultrapure water.

RNA extraction was conducted in biological triplicates from spikelets from the three cultivars, collected following the same criteria used to obtain the samples for library construction. Briefly, spikelets with basal flowers at the beginning of anthesis were collected based on the reproductive calendar shown in Selva et al. (2017), where all developmental stages are represented, from early archespores to mature female gametophytes, evaluated through observations under a stereoscopic microscope (Leica S APO) of several collected spikelets. RNA isolation was performed using a TRIzol based protocol. Approximately 35 mg of tissue from biological triplicates per sample was frozen and homogenized in liquid nitrogen using a plastic pestle in a 1.5 ml Eppendorf tube. Then, they were mixed with TRIzol™ Reagent (Invitrogen). Following the addition of chloroform, RNA was precipitated from the upper layer with isopropanol and washed with ethanol. The samples were resuspended in 20–25 μl of DEPC water and stored at −80°C. RNA was quantified in a DeNovix DS-11 spectrophotometer (Denovix Inc., USA), whereas its integrity was checked by agarose gel electrophoresis.

### cDNA and stem-loops based cDNA synthesis

RNA was used for the synthesis of cDNA, as well as stem-loop cDNA. The cDNA synthesis was performed aiming for the validation of transcripts’ differential expression, using the ImProm II™ Reverse Transcription System (Promega) following the supplier’s instructions.

Stem-loop synthesis was aimed at miRNA quantification (Chen et al., 2005), and was also conducted using the ImProm-II™ Reverse Transcription System, but in this case instead of using random primers, a specific stem loop of ≈50 bp was designed for each of the miRNAs intended to be validated and quantified. Stem-loop primers were designed manually, as described in Garbus et al. (2019) (**Table 1**).

**Table 1:** Primers designed for stem-loop miRNAs amplification.

Primer	Sequence	Size (bp)	tm (°C)
mat_810_DREB2	TGTGTTCTCAGGTCGCCCCCG	21	70.9
ST_mat_810_DREB2	GTCGTATCCAGTGCAGGGTCCGAGGTATTCGCACTGGATACGACcggggg	50	80.5
mat_42_KINESIN	GTGTTCTTTGCATCTTGGTCAAGC	24	60.7
ST_42_KINESIN	GTCGTATCCAGTGCAGGGTCCGAGGTATTCGCACTGGATACGACgcttga	50	76.7
mat_422_PATATIN	AGGTGATTTTTGGATTATGTTCAG	24	55.0
ST_mat_422_PATATIN	GTCGTATCCAGTGCAGGGTCCGAGGTATTCGCACTGGATACGACctgaac	50	76.5
mat_181_DdrPOL	GGAATGTTGTCTGGTTCAAGG	21	59.2
ST_mat_181_DdrPOL	GTCGTATCCAGTGCAGGGTCCGAGGTATTCGCACTGGATACGACccttga	50	77.4
mat_676_GAMYB	AGAGTTGGAGGAAAACAAACC	21	58.1
ST_mat_676_GAMYB	GTCGTATCCAGTGCAGGGTCCGAGGTATTCGCACTGGATACGACggtttg	50	76.5
mat_601_SQUA	TGACAGAAGAGAGTGAGCAC	20	58.7
ST_mat_601_SQUA	GTCGTATCCAGTGCAGGGTCCGAGGTATTCGCACTGGATACGACgtgctc	50	77.1
mat_750_GRF8	TCCACAGGCTTTCTTGAACTG	21	60.1
ST_mat_750_GRF8	GTCGTATCCAGTGCAGGGTCCGAGGTATTCGCACTGGATACGACcagttc	50	76.5
URP	CCAGTGCAGGGTCCGAGGT	19	69.3

Reactions were conducted from 20 ng of RNA, in biological triplicates for OTA-S, Tanganyika and Don Walter. Briefly, each 20 μl reaction was incubated in a BioRad Thermocycler for 5 min at 25°C followed by 60 min at 42°C and finally 15 min at 70°C to inactivate the reverse transcriptase, and cooled at 4°C.

### PCR and RT-PCR

Primers based on the sequence of the transcripts differentially regulated among libraries (**Table 2**), were designed using the IDT Interphase (https://www.idtdna.com/PrimerQuest/Home/Index).

**Table 2:** Transcripts and/or gene based primers, designed for PCR and qPCR amplification.

Primer	Sequence	Size (bp)	Tm (°C)
GAMYB_F1	CCACTTCCGTCTCTGATCTTCT	22	61.4
GAMYB_R1	TCCGGGCGAAGGACTTG	17	64.4
GAMYB_F2	GGGCATGATGGCAGAGAG	18	61.4
GAMYB_R2	GCTCATAAATCAGTTCAGAGAATCATAG	28	56.4
GAMYB_RT_F3	CGCGGTGCAGAAGATGAG	18	61.0
GAMYB_RT_R3	CCTCGTGTTCCAGTAGTTCTTG	22	60.2
SQUA_F1	ATGGACCGCAAGGACAAGTC	20	62.8
SQUA_R1a	CGCCTCGCACACCTTGT	17	63.3
SQUA_R3	CTCCTCTTGATGTCGTCGAAC	21	60.4
SQUA_F4	GGAGTTCGACGACATCAAGAG	21	60.4
SQUA_R4	ATGTGTGAGAAACTTTACTACTTGC	25	55.9
SQUA_RT_Fe5	CCAAGCGCTACCACAAGA	18	59.6
SQUA_RT_R5	CTCCGACAGCTCATGGAAC	19	61.0
PAT_F1	CACTCAGATCAGAAGCACAAGA	22	58.7
PAT_R1	CGGTGCCAATGGAGATGAT	19	60.2
PAT_F2	GTAACCCGGATTTCAACCCT	20	60.6
PAT_R2	AGTGATCATCTTCCAACAACCT	22	58.2
GRF8_F1	AACTGTAATCCTGGACGTTTCA	22	58.2
GRF8_R1	AGGAGCATTCGCGACAAA	18	58.7
GRF8_F2	GCACCAGGCCCTGATCTA	18	62.7
GRF8_R2	GTGTGGCAGTGCGATGATAC	20	60.0
GRF8_RT_F4	TCAATCCGTGGAGTACCTTTG	21	59.7
GRF8_RT_R4	GTAGCGACCACGGTTTAAGT	20	58.8
DdRPolII_F3	CATACTGAAACCAAGGCCTATTTG	24	57.9
DdRPolII_R3	CATTGTCGAACTCACCCTTCT	21	59.7
DREB2_F2	GTGCTCTTCCCACGAATGA	19	60.1
DREB2_R2	ACCCTTCTTGGAACCCTTTG	20	61.4
DREB2_RT_F4	GGCCTCTAATCTGGGAAAGAAG	22	60.7
DREB2_RT_R4	CCTCACGCCACGGAATC	17	62.2
KIN_F1	GGAGGCCACCGACGACG	17	68.4
KIN_R1	GTGCCCAGCCTGAGTAGAGATA	22	62.9
KIN_F6	CGGTGGTGCAGAAACTGATA	20	59.4
KIN_R6	CGGACATCATGCCAGAAAGA	20	60.0

Amplification of the genomic sequence of the corresponding transcripts, as well as the miRNA precursors, was conducted using DNA. The reactions were run using a MyCycler Thermal Cycler (Bio-Rad, Hercules, CA, USA), and prepared with 0.3 μl of 40 mM dNTPs mix, 3 μl of 10× reaction buffer, 0.6 μl of each forward and reverse primer (10 pmol/μl), 0.2 μl of DNA polymerase, 1 μl of template genomic DNA (40 ng/μl), in a final reaction volume of 15 μl. The DNA polymerases used were either Taq Pegasus (PB-L) or GoTaq DNA Polymerase (Promega), both containing 5U enzyme/μl. The protocol followed for DNA amplification consisted of an initial DNA denaturation step at 95°C for 2-5 min, followed by 33 cycles at 95°C for 30 s, 30 s at the optimal annealing temperature for each primer pair, and 72°C for 30s. The final step consisted of a final extension of 3 min at 72°C. The primer annealing temperatures were optimized for each primer pair, starting from one degree below the lower melting temperature between both primers.

RT-PCR reactions were performed to verify and visualize the amplification among genotypes of the differentially regulated transcripts predicted *in silico*. Reactions were developed basically as described for the PCR ones, but starting from 2 μl of a 1/20 cDNA dilution.

PCR and RT-PCR amplification bands were separated by electrophoresis in a 1.5% (m/v) agarose gel and visualized using ethidium bromide in a VP High Performance UV Transilluminator.

### Stem-loop miRNA amplification

The stem-loop miRNA amplification was performed from the cDNA amplified from the specific stem-loop sequences for each miRNA, using the miRNA sequences as the forward primers and an URP primer (Universal Reverse Primer), based on the stem-loop sequence, as the reverse primer (**Table 1**). The amplification reactions were conducted essentially as described for PCR, but starting from 2 μl of a 1/20 dilution of the retrotranscription reaction already described.

### miRNAs precursor amplification

Specific primers for pri-miRNAs and pre-miRNAs were designed based on Don Walter genomic sequences, predicted by the mirDP2 software (**Table 3**). The amplification was carried out using a different forward primer for pri-miRNA and pre-miRNA, and the same reverse primers for both.

**Table 3:** Primers designed based on miRNA precursors identified in the Don Walter genome.

Primer	Sequence	Tm (°C)
pcs_pri_mat181F	GTGGAATGTTGTCTGGTTCAAG	58.9
pcs_pre_mat181	GGAATGTTGTCTGGTTCAAGG	59.2
pcs_rev_mat181	GAATGAAGCCTGGTCCGA	60.6
pcs_pri_mat42F	ACTTGCTAAGGAAGATCGTAGTG	57.7
pcs_pre_mat42F	GTGTTCTTTGCATCTTGGTCA	57.6
pcs_rev_mat42	TAGCATTCAAACACTTGAGCAC	56.6
pcs_pri_mat422F	AGATTGAACCTAAGCTTAGATA	51.0
pcs_pre_mat422F	GGTGATTTTTGGATTATGTTCAG	54.0
pcs_rev_mat422	AAGTTCTAGGCACATTGGC	56.6
pcs_pri_mat676F	ACTGTTATGACTAGGTTCTATC	51.9
pcs_pre_mat676	AGAGTTGGAGGAAAACAAACC	58.1
pcs_rev_mat676	TGAGATATTGGAGAAAAACAAG	52.1
pcs_pri_mat750F	ATGCTCTCCACAGGCTTTC	59.6
pcs_pre_mat750F	TCCACAGGCTTTCTTGAAC	57.7
pcs_rev_mat750	GGACTTTCTTGAACCATCAACAC	58.9
pcs_pri_mat810F	GGCGAGCTGCGAACACAT	62.4
pcs_pre_mat810F	GGGGCGAGCTGCGAACACATG	68.6
pcs_rev_mat810	CGGGGGCGACCTGAGAACACA	70.9
pcs_pri_mat601	CGGTGATTTCCATGGCTAACT	59.5
pcs_pre_mat601	TGACAGAAGAGAGTGAGCAC	58.7
pcs_rev_mat601	ACTGACAGAGAGAGAAGTGAGC	60.4

### qPCR reactions

The experiments carried out to validate the differential regulation of transcripts and miRNAs described *in silico* for the three genotypes, were conducted through Quantitative Real-Time PCR (qPCR), using the thermocycler CFX Connect Real-Time PCR System (Bio-Rad). Reactions were performed in a final volume of 20 ul, and contained 50 pmol of forward and reverse primers, 3 µl of 200-fold diluted cDNA and 10 µl of Real Mix (Biodynamics, Argentina). The *E. curvula* housekeeping gene UBICE (Ubiquitin conjugating enzyme transcript) was used to normalize the specific gene expression levels, as in Selva et al., 2020. The thermal cycling used for these amplifications consisted of 95°C for 2 min, 40 cycles at 94°C for 10 s, 15 s at the optimal annealing temperature for each primer pair and finally at 30s at 72°C. Reactions were conducted on technical triplicates of biological duplicates for the three assayed genotypes. Dissociation curve profiles were used to verify the specificity of each reaction. Normalization of transcript levels between cDNA samples was undertaken using the 2-ΔΔCt method (Livak and Schmittgen, 2001). The statistical analysis of the qPCR fold change in the expression of genes among different treatments was done through the Student’s t-test, where a p-value of 0.05 was considered to be significant.

## Results

### Don Walter small RNA libraries characterization

**Figure 1:**
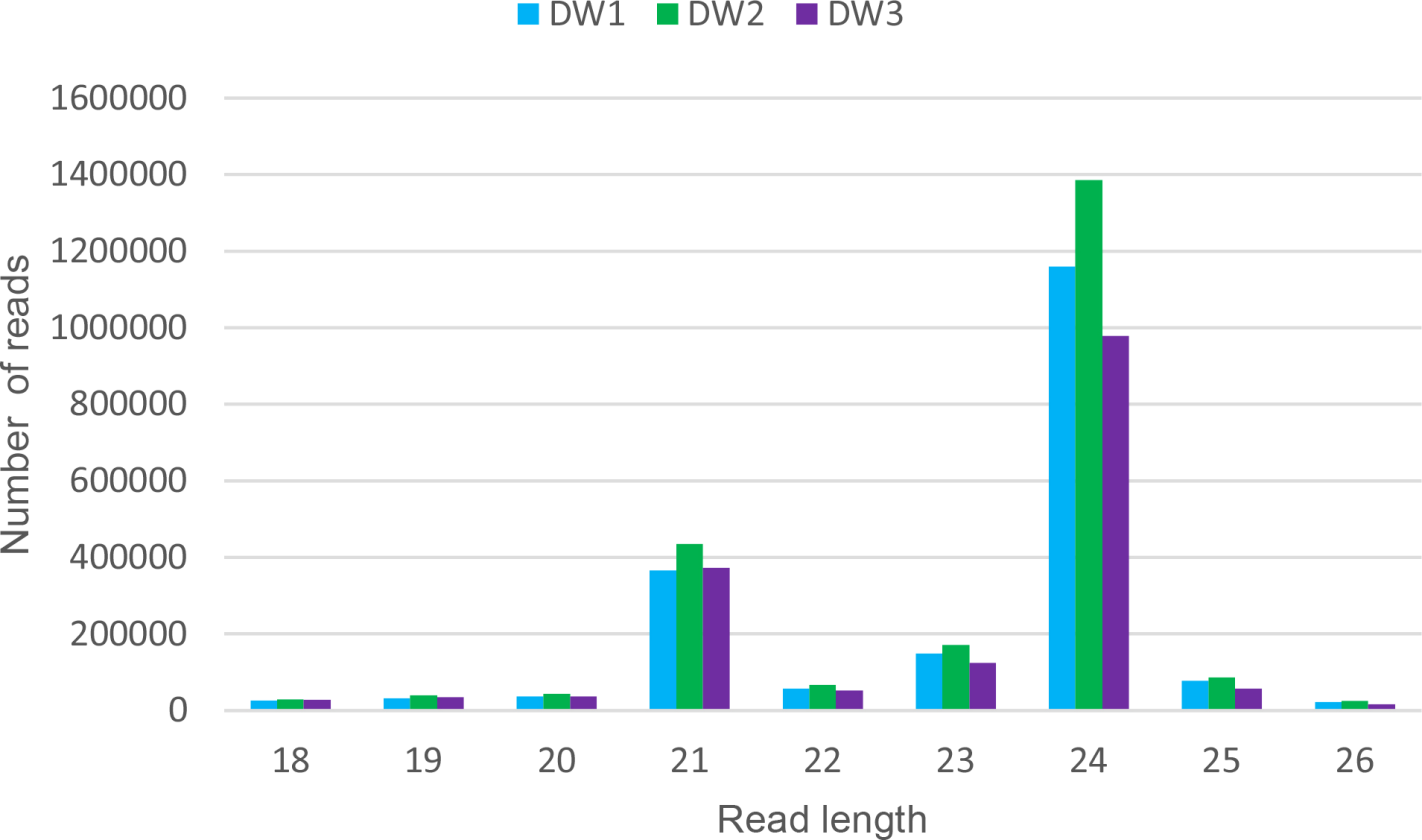
Read length distribution of the three Don Walter sRNA libraries. For each read length, the absolute read count per individual library is shown. The x-axis shows read lengths, and the y-axis shows the frequency of occurrence of reads of each length. The over-representation of 21 and 24 bp reads is evidenced.

Small RNAs libraries from inflorescences of the cultivar Don Walter were constructed for this work, whereas those from Tanganyika and OTA-S have been reported in Garbus et al., 2019. Thus, we started from a brief characterization of Don Walter sRNA libraries. There were obtained 2,541,355 raw reads for DW1, and 2,929,269 and 2,140,845 for DW2 and DW3 respectively. After trimming, a total of 2,114,363 (83.1%), 2,556,295 (87,2%) and 1,802,287 (84,1%) reads were retained for each library. As expected, reads with 21 and 24 bp were overrepresented in the three samples (**Figure 1**).

### miRNA prediction and expression

To identify miRNA sequences and its precursors, the reads from the seven samples, i.e., three from the facultative Don Walter, two from the sexual OTA-S and two from the full apomictic Tanganyika cultivar, were merged in a single file. After trimming and filtering, a total of 171,339,858 reads passed the quality control. Since miRDP2 pipeline requires unique sequences, the input file was collapsed into 30,124,624 unique reads attaching their frequency to the header.

**Figure 2:**
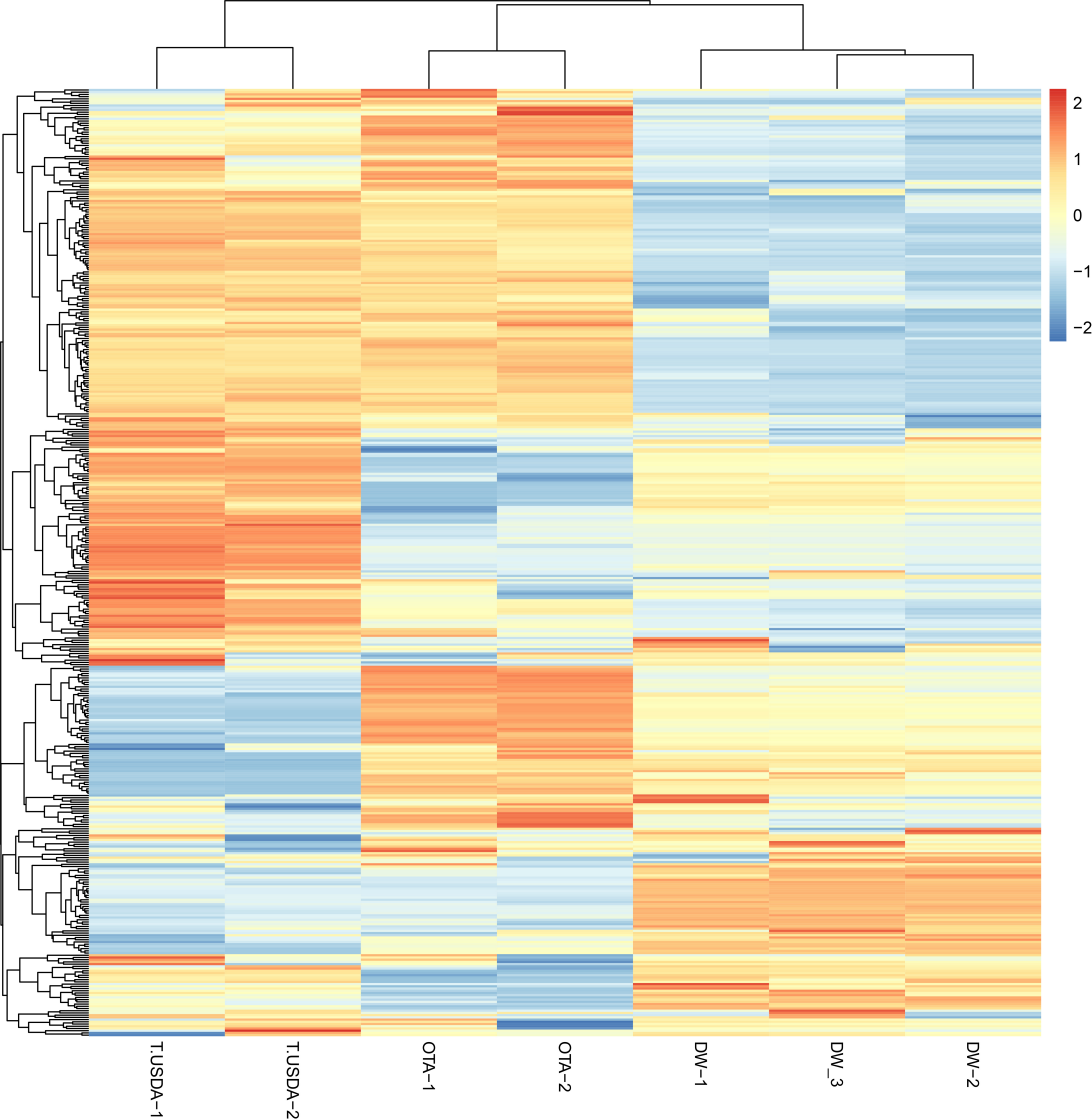
Heatmap showing the expression of the miRNAs in the seven samples analyzed. The count matrix was transformed in the log2 scale and normalized according to the library size. The considered samples are shown in the x-axis. In the color scale, orange and blue represent up-and down-expressed miRNAs respectively.

The miRDP2 software predicted 953 miRNAs that were used as reference to map each sample through the Bowtie software. A count matrix was constructed to quantify the expression of the mature miRNAs in the samples. After filtering, a PCA plot and a heatmap were constructed using the normalized matrix as input, showing the similarity within samples with the same reproductive behavior (**Supplementary Figures 1**, **2**). Finally, the same matrix was used to show the expression of all the miRNAs in each sample (**Figure 2**). The differential expression analysis showed that 51, 108 and 51 mature miRNAs were up-regulated in APOvsSEX, APOvsFAC and FACvsSEX comparisons, respectively. On the other hand, the down-regulated miRNAs were 52, 57 and 99 for the APOvsSEX, APOvsFAC and FACvsSEX, respectively.

### Target identification and transcript expression analysis

Target identification was performed through psRNATarget software (Dai et al., 2018) using the improved scoring schema V2 (2017). A total of 13,770 targets were found, which were putatively regulated by 682 unique mature miRNAs. In parallel, the expression of the transcripts was assessed. The reads were mapped against the transcripts using hisat2 (Kim et al., 2019) in order to quantify its expression. The differential expression analysis showed that 220, 2,162 and 542 transcripts were upregulated for the APOvsSEX, APOvsFAC and FACvsSEX, respectively; whereas the downregulated transcripts for the same comparisons were 195, 798 and 1,520, respectively.

### Transcripts regulated by miRNAs

Based on the psRNAtarget results, we focused especially on those possible transcript-miRNA interactions in which the differential expression of transcripts and miRNAs among genotypes followed opposite direction, i.e., when transcripts showed to be up-regulated in a particular comparison, the miRNA resulted down-regulated and *vice versa*. Hence, 5, 14, and 16 target transcripts were found to be down-regulated while its mature miRNA was found to be up-regulated in the APOvsSEX, APOvsFAC and FACvsSEX comparisons, respectively. On the other hand, 14 and 6 targets were found up-regulated for the APOvsFAC and FACvsSEX comparison, while the mature miRNAs were found to be down-regulated. No miRNA-mRNA interactions were identified for the comparison APOvsSEX.

### Genomic DNA and transcript expression analysis

The *in silico* analysis revealed the existence of ≈60 possible miRNA-target interactions that could be involved in the regulation of the reproductive mode in *E. curvula* (**Supplementary Table 1**). For validation purposes, after transcript annotation, seven miRNA-target interactions were selected mainly guided by their possible known function, discarding in this initial approach those transcripts without annotation and/or corresponding to hypothetical proteins (**Table 4**). Thus, for the comparison APOvsFAC with the target down-regulated and the miRNA up-regulated, the expression of the genes *Kinesin-like protein KIN-14D*, *Patatin-like protein 2* and *Dehydration-Responsive Element Binding Protein 2A* (DREB2A) (**Supplementary Figure 3A–C**) was explored, whereas for the target up-regulated and miRNA down-regulated combination, the expression of *Growth-regulating factor 8* (GRF8) and *Squamosa-promoter binding protein-like protein 13* genes was inquired (**Supplementary Figure 3D, E**). The expression of the genes for *DNA-Directed RNA polymerase II subunit RPB1* (DRdRNApolII) and *GAMYB transcription factor* were investigated because they were differential for the comparison FACvsSEX, with the target being down-regulated and miRNA up-regulated (**Supplementary Figure 3F, G**).

**Table 4:** **miRNA-mRNA interactions selected for the analysis**.

	miRNA	Target	Transcript	Size (bp)	Protein	ID	Size (AA)	miRNA from the libraries	Transcript target site	Conserved miRNA	*E. curvula* miRNA
**APOvsFAC**	Up- regulated	Down- regulated	maker-Backbone_4151-snap-gene-0.33	4062	Kinesin-like protein KIN-14D	Q0E2L3.2	977	miRNA_42	371-394	-	Ecu-miRNA_novel_1
			maker-Backbone_1301- augustus-gene-1.1	1699	Patatin-like protein 2	A2YW91.1	405	miRNA_422	310-084	-	Ecu-miRNa_novel_2
			maker-Backbone_2520- snap-gene-0.20	1737	DREB2A	JAF93766	343	miRNA_810	532-552	Zma-miR398b	Ecu-miR398
	Down- regulated	Up- regulated	augustus_masked-Backbone_566-processed-gene- 1.5	2073	Growth-regulating factor 8	Q6AWY1.1	409	miRNA_601	719-738	Zos-miR156e	Ecu-miR156c
			maker-Backbone_1343-snap-gene-1.63	1093	Squamosa-promoter binding protein-like protein 13	AQQ11858	195	miRNA_830	719-739	Tae-miR156a	Ecu-miR156d
**FACvsSEX**	Up- regulated	Down- regulated	maker-Backbone_4804-snap-gene-0.1	3972	DNA-directed RNA polymerase II subunit RPB1	P18616.3	1839	miRNA_181	3540-3560	Osa-miR166e	Ecu-miR166
			maker-Backbone_7431-snap-gene-0.35	2017	Transcription factor GAMYB	OEL28920	432	miRNA_676	2081-2101	Osa-miR2275b	Ecu-miR2275c

The transcripts identified in the Don Walter transcriptome that resulted up or down regulated from the comparisons APOvsSEX and FACvsSEx were annotated as well as the miRNAs that targeted them, when they were conserved. Protein name and ID were retrieved from the UNIport database.

**Figure 3:**
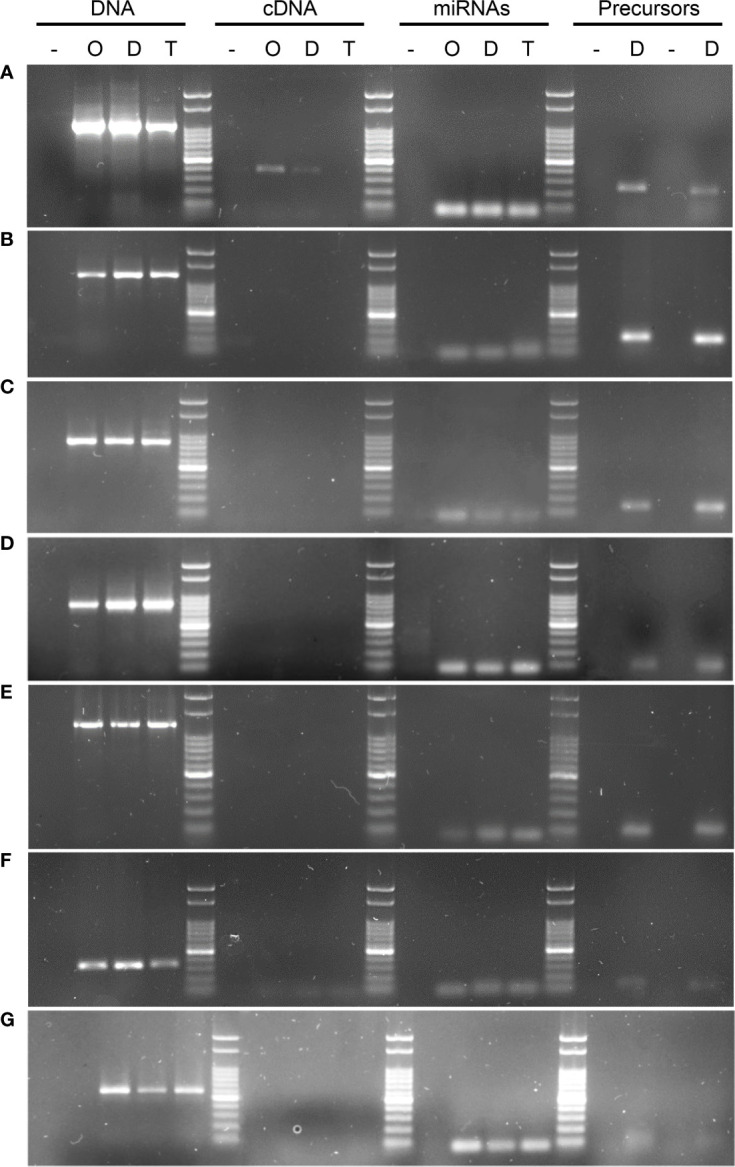
PCR and RT-PCR amplifications using gene, transcript, miRNA, pri-miRNA and pre-miRNA based primers. The image shows DNA from the target genes could be amplified in all of the tested samples, whereas when cDNA was the template, this led to amplification bands for 1 of the 7 transcripts tested using several primers combinations (**Table 5**). **(A)** KINESIN, which showed amplification in cDNA, and was absent only in Tanganyika, **(B)** PATATIN, **(C)** GRF8, **(D)** DREB2, **(E)** DdRNAPolII1B, **(F)** GAMYB, **(G)** SQUAMOSA. *UBICE* gene was used for normalization of cDNA expression. The amplification using miRNA, pri-miRNA and pre-miRNA based primers allowed the validation of the novel and reported miRNAs tested in this study.

Initially, PCR reactions were conducted aiming to amplify the mentioned genes from genomic DNA for the three cultivars in biological triplicates, leading to the predicted amplification bands, using one or more specific primer combinations (**Table 5**, **Figure 3**). Interestingly, the kinesin transcript was differentially expressed among genotypes, being absent in the apomictic cultivar Tanganyika, in accordance with the prediction of being down-regulated in the APOvsSEX comparison (**Figure 3A**). However, the primer combination used for the other transcripts resulted in no amplification bands (**Table 5**, **Figure 3B–G**). In order to confirm the absence of expression, qPCR reactions were conducted. UBICE was amplified from all the cDNA samples with no significant difference in expression among them. However, the Cts obtained for the assayed cDNA samples were close to that of the negative controls, indicative of the absence of expression. Kinesin was not subjected to qPCR, since the observed amplification bands were conclusive.

**Table 5:** PCR amplicons expected and predicted sizes obtained from DNA and cDNA with several combinations of transcript-based primers.

	miRNAs	Target	Target gene	Forward primer	Reverse primer	Expected DNA amplicon size (bp)	Obtained DNA amplicon size (bp)	Expected cDNA amplicon size (bp)	Obtained cDNA amplicon size (bp)
**APOvsFAC**	Down- regulated	Up- regulated	Squamosa-promoter binding protein-like protein 13	SQUA_F1	SQUA_R1a	x	(-)	320	(-)
				SQUA_RT_F5	SQUA_R3	x	(-)	141	(-)
				SQUA_F4	SQUA_R4	572	~600	572	(-)
				SQUA_RT_F5	SQUA_RT_R5	x	(-)	121	(-)
			Growth-regulating factor 8	GRF8_F1	GRF8_R1	1028	~1000	580	(-)
				GRF8_F2	GRF8_R2	1028	~1000	350	(-)
				GRF8_RT_F4	GRF8_RT_R4	853	~850	175	(-)
	Up- regulated	Down- regulated	Patatin-like protein 2	PAT_F1	PAT_R1	1131	~1150	916	(-)
				PAT_F2	PAT_R2	931	~950	749	(-)
			Dehydration-Responsive Element Binding Protein 2A (DREB2A)	DREB2_F2	DREB2_R2	834	~850	229	(-)
				DREB2_RT_F4	DREB2_RT_R4	x	(-)	221	(-)
			Kinesin-like protein KIN-14D	KIN_F1	KIN_R1	1076	~1100	636	(-)
				KIN_F6	KIN_R6	1048	~1050	300	O, DW: 300; T: (-)
**FACvsSEX**	Up- regulated	Down- regulated	Transcription factor GAMYB	GAMYB_F1	GAMYB_R1	1165	~1150	1062	(-)
				GAMYB_F2	GAMYB_R2	1317	multiple bands	363	(-)
				GAMYB_RT_F3	GAMYB_RT_R3	322	~300	219	(-)
			DNA-directed RNA polymerase II subunit RPB1	DdRPolII_F3	DdRPolII_R3	1204	~1200	1009	(-)

A total of 7 miRNA–target combinations that arise from the APOvsFAC and FACvsSEX comparisons with opposite miRNA/target regulation have been tested.

### Validation of novel and conserved miRNAs

**Figure 4:**
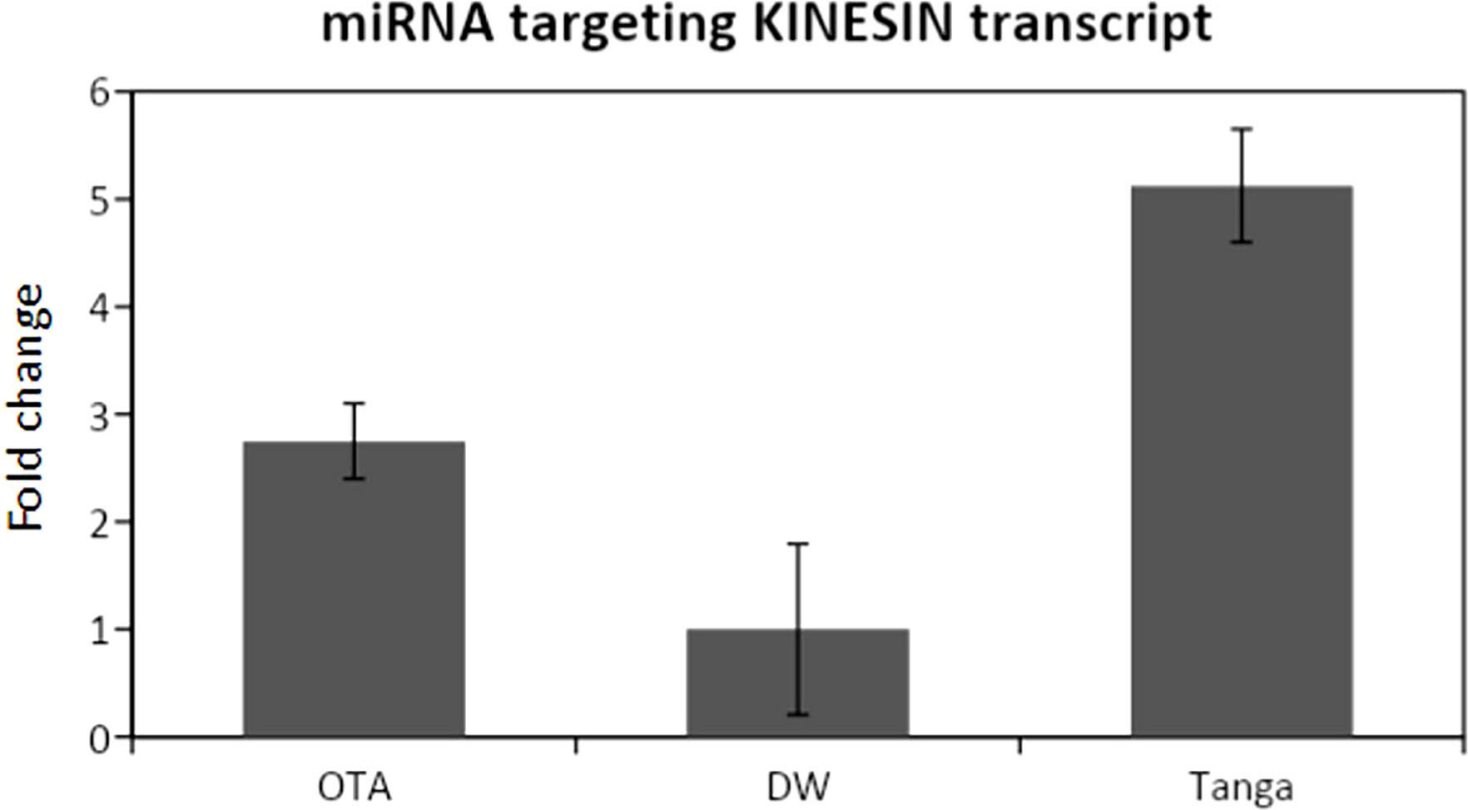
qPCR analysis for the miRNA that targets the Kinesin transcript. DW: Don Walter. Tanga: Tanganyika. The miRNA is overexpressed in the apomictic cultivar Tanganyika (p<0.05). Data was normalized with respect to the genotype that showed the lower expression.

The expression of the miRNA predicted to be interacting with the mentioned transcripts was analyzed through RT-PCR and qPCR, revealing that all the miRNAs were expressed, whereas differential expression was only detected from the miRNA that targets the kinesin transcript (**Figure 4**).

**Figure 5:**
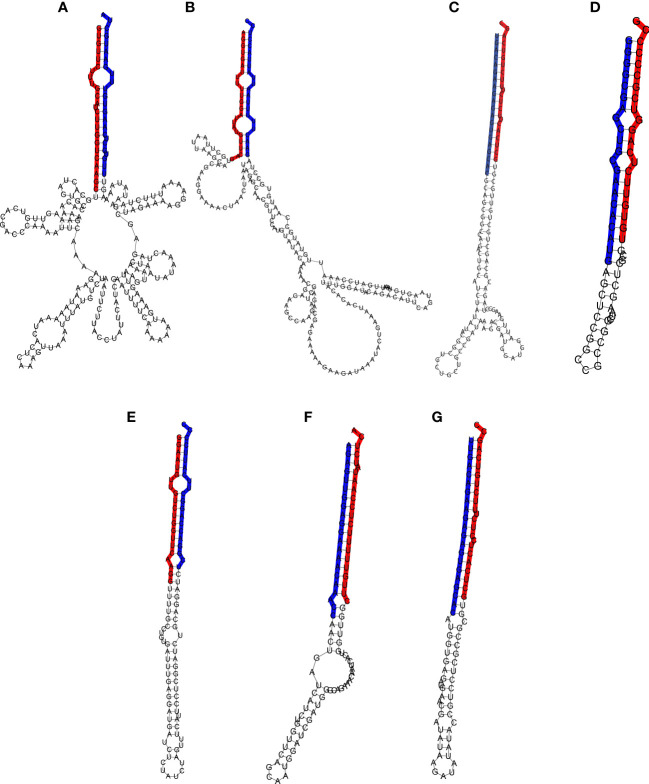
Hairpin structure of the miRNA precursors. The mature and star sequences are highlighted in blue and red, respectively. **(A)** Pre-Ecu-mir novel_1, **(B)** Pre-Ecu-mir novel_2, **(C)** Pre-Ecu-mir156c, **(D)** Pre-Ecu-mir398, **(E)** Pre-Ecu-mir166, **(F)** Pre-Ecu-mir 2275c**, (G)** Pre-Ecu-mir156d (**Table 4**).

miRNAs were further validated through the amplification from DNA of their pri-miRNA and pre-miRNA precursors, predicted from the Don Walter genome (**Figure 3A–G**), whereas their hairpin structures are shown in **Figure 5**. The sequences of the mature and star miRNAs as well as their precursor are reported in **Supplementary Table 2**.

The expression of all the tested miRNAs, as well as their precursors and the validation of their hairpin structures, together with the absence of amplification for most of the searched transcripts strongly suggests that a miRNA-target regulation exists for all the combinations tested.

## Discussion

*Eragrostis curvula* (Schrad.) Nees, commonly known as weeping lovegrass, is a perennial grass from the Poaceae family, widely cultivated in the semiarid region of Argentina as a forage grass (Covas, 1991). *E. curvula* has a basic chromosome number of x=10, and it includes genotypes with different ploidy levels. While the diploid cytotypes are scarce and sexual in nature, tetraploid plants and plants with higher ploidy levels reproduce by pseudogamous diplosporous apomixis, defined as asexual reproduction through seeds (Leigh, 1960; Voigt and Burson, 1983; Noirot et al., 1997). Apomixis has been described in over 400 angiosperms, and it leads to the creation of clones of the mother plant (Nogler, 1984). However, this attribute is absent in most species of agricultural importance. The study of the genetic regulation of this mechanism would be important for the development of tools that could enable its transference to key species, such as wheat, rice or maize, in order to fixate important agronomic traits through generations (Hanna and Bashaw, 1987). The purpose of this work was focused on analyzing the epigenetic mechanisms that could be playing a role in diplosporous apomictic reproduction in this species.

Studies suggest that, instead of an independently originated trait, apomixis arises as the result of modifications in the temporal or spatial regulation of the sexual pathway in seed development, which produces variations or omissions in critical developmental stages (Koltunow and Grossniklaus, 2003; Schmidt, 2020). With this theory in mind, the expression of a gene or genes in specific cells from the nucellus could determine if sexual or apomictic processes, or both, will take place in that ovule, proposing that apomixis-related genes must be absent or unexpressed for the sexual processes to take place (Koltunow, 1993; Koltunow and Grossniklaus, 2003).

Genomic and transcriptomic studies were performed in the past to discover the molecular mechanisms involved in apomixis. Even though several advances were made in the last years, candidate genes related to meiosis could not be found through these approaches so far. Moreover, the involvement of epigenetic factors is being studied, as they can occur rapidly and are particularly frequent as a result of polyploidization and hybridization, and they could also help explain the parallel and independent occurrence of apomixis several times during evolution (Koltunow and Grossniklaus, 2003; Comai, 2005). In the year 2010, García-Aguilar et al., 2010 observed that a deregulation of DNA methylation produced apomeiotic-like phenotypes in reproductive cells of maize. Also, in apomictic genotypes of Boechera subjected to stress, Taraxacum-type diplospory arised in the female gametophyte. In studies with the same genotypes, the suppression of DNA methylation before the formation of the MMC triggered Antennaria-type mitotic diplospory (Gao, 2018). In *E. curvula*, it has been observed that environmental signals have an influence over the number of sexual embryo sacs (Rodrigo et al., 2017), and that polyploidization involves an increase in genome methylation, and it could lead to gene silencing, in order to preserve the functional characteristics of the diploid (Ochogavía et al., 2009; Zappacosta et al., 2014; Carballo et al., 2021).

As mentioned previously, miRNAs are known to play central regulatory roles during plant development, and they are part of diverse biological processes such as flower and leaf morphogenesis, response to stress, reproductive cell identity, fertility, etc. (Aukerman and Sakai, 2003; Palatnik et al., 2003; Sunkar and Zhu, 2004; Schwab et al., 2005; Ru et al., 2006; Olmedo-Monfil et al., 2010; Amiteye et al., 2011; Li et al., 2015; Ripoll et al., 2015; Fei et al., 2021).

Our work established that the gene *kinesin* is differentially expressed among genotypes, being absent in the apomictic genotype Tanganyika. A BLAST analysis of the transcript revealed that it is homolog of Kinesin14D. Moreover, the expression of the miRNA postulated to be regulating kinesin, was found to be upregulated in the genotype Tanganyika. This is a novel miRNA identified in this work, referred in the manuscript Ecu-miRNA_novel_1, and its existence was confirmed by the amplification of the precursors pri-miRNA and pre-miRNA and the predicted hairpin structure. Kinesins act as microtubule-based molecular motors involved in several biological processes such as cell division, intracellular transport, and cell shape determination (Zhu and Dixit, 2012; Ali and Yang, 2020). *Kinesin* genes are distributed in 14 families, with Kinesin7 and Kinesin14 being the most represented in land plants (Richardson et al., 2006). It is proposed that land plants may have evolved novel Kinesin14 motors to account for microtubule-based functions that are plant-specific or are typically performed by Dynein in other eukaryotes, as is spindle formation and the expansion of the phragmoplast (Zhu and Dixit, 2012; Gicking et al., 2018).

The Kinesin14 family includes many members that are up-regulated during mitosis and function during cell division and localization of plastids, thus influencing plant development (Gicking et al., 2018). ATK1 and ATK5 are two minus-end-directed Kinesin14 proteins that localize in the spindle apparatus. *A. thaliana atk1* mutants exhibit abnormalities in meiosis, such as extended spindle poles and reduced male fertility (Chen et al., 2002), as well as mild spindle bipolarity defects during mitosis (Marcus et al., 2003). The lack of spindle bipolarity in *atk1-1* leads to abnormal chromosome segregation during meiosis, whereas in mitosis this defect is corrected by anaphase, perhaps due to functional compensation by other minus-end-directed kinesins such as ATK5 (Nebenführ and Dixit, 2018). Mitotic spindles in the *atk5-1* mutant are abnormally broad (Ambrose et al., 2005). Furthermore, maize mutants in a Kinesin14 member, that is likely a functional homolog of ATK1, showed extended spindle poles and altered spindle length and width during meiosis (Higgins et al., 2016).

A study conducted in *Citrullus lanatus*, showed that 38 Kinesin genes were overexpressed in the reproductive organs, being 20 preferentially expressed in the female flower, and 9 specifically expressed in the male flowers, thus suggesting that they play critical roles in the growth and development of reproductive tissues (Tian et al., 2021). Interestingly ClKIN14D, homologous to Kinesin14D, was found to be overexpressed in male flowers and repressed in female flowers (Tian et al., 2021).

The analysis of protein motifs of the Kinesin translated from Don Walter transcriptome, revealed that it contains a calponin-homology domain (CH), thus classified as KCH, a unique subset of Kinesin14 that can bind to both microtubules and actin filaments in land plants (Umezu et al., 2011). KCHs are most abundant in developing tissues and are thought to participate in cell division and elongation (Frey et al., 2010). Identified KCHs showed to be mostly expressed in mature pollen, being proposed that they are the main actors for the movement of sperm cells in the pollen tube (Schattner et al., 2021). The expression of OsKCH1 was quantified in rice, revealing that the highest abundance of transcripts was found in primary leaf, primary root, and the developing flower (Frey et al., 2010).

In this bibliographic context it is clear that kinesins are involved in plant reproduction and, thus, is plausible that the miRNA-target interaction that impedes the translation of the kinesin transcript is contributing to a defective sexual expression and leading to an apomictic phenotype in Tanganyika. Moreover, Kinesins were reported to be targeted by miR1863 (Smita et al., 2021) in grapevine and by sly-miR171a in *Solanum lycopersicum* (López-Galiano et al., 2019). It is not unexpected that kinesins, with a main role in mitosis and meiosis, are determinant in plant growth and fertility.

The expression of the orthologous gene from Arabidopsis, At1g73860, was demonstrated to be tissue-specific for the microgametogenesis, specifically expressed in mature pollen (Honys and Twell, 2004; Klepikova et al., 2016), thus arguing in favor of a specific role in the reproductive pathway. In addition, the rice orthologous Os02g0229600, was found highly expressed in mature pollen as well as in pre-flowering panicles (Sakai et al., 2011).

Whether the epigenetic mechanisms are the cause or the result of changes in the reproductive behavior is still under discussion (Hand and Koltunow, 2014; Mau et al., 2022), but under the light of our results, the fact that the kinesin gene is involved in apomixis in *E. curvula* cannot be denied. Thus, this gene is disclosed as a strong candidate for further studies allowing the design of strategies to inquire into apomixis mechanisms in this or other species.

Concerning the other analyzed genes, the absence of mRNA expression combined with the observed miRNA expression demonstrated miRNA-target interactions for all of them. Out of the analyzed transcripts, DNA-directed RNA polymerase II subunit RPB1 (DdRNAPolII1B), Squamosa-promoter binding protein-like 13 (SBP13), Growth-regulating factor 8 (GRF8) and Dehydration-Responsive Element Binding Protein 2A (DREB2A) are targeted by the conserved miRNAs with identity to Osa-miR166e, Tae-miR156a, Zma-miR396d and Zma-miR398b respectively, whereas the other transcripts are regulated by miRNAs firstly described in this work, and validated through the amplification of the pri-miRNAs and pre-miRNAs (**Table 4**).

Interestingly, several of our analyzed transcripts were previously demonstrated to be regulated by miRNAs. miR156 was reported to target SBP genes (Poethig, 2010; Wang, 2014; Garbus et al., 2019), and was shown to be highly conserved, playing an important role in plant development (Poethig, 2010). It is highly expressed during the juvenile phase and declines during vegetative phase change (Poethig, 2010), leading to an increased expression of SBPs genes that act by promoting the development of adult vegetative traits and floral induction (Wang, 2014). Overexpression of miRNA156 in *A. thaliana* produces nearly full sterility, and the rescue of SPL function brings back normal reproductive behavior (Zheng et al., 2019). It is also known that miRNA156’s expression is fine-tuned since its temporal and spatial expression change during the reproductive development (Wu and Poethig, 2006; Zheng et al., 2019). We can hypothesize that the expression of miRNA156 could have different expression patterns related to the reproductive mode. Additional analyses testing its expression in different developmental stages of apomictic and sexual plants should be conducted to confirm this assumption.

The transcription factor GAMYB (Gibberellin-and-Abscisic acid-regulated MYB), involved in flowering time and flower organ development (Sunkar and Zhu, 2004), was also reported to be involved in another development *via* regulating the expression of phenylalanine ammonia lyase (PAL) (Woodger et al., 2003). It was proposed that miR319a-regulated GAMYB might also be involved in the browning inhibition of fresh-cut apples by H2S treatment (Chen et al., 2020). Other study states that, in *Oryza sativa*, miR1026 and miR159 target DdRNAPolII1B and GAMYB, respectively, being both up-regulated under stress conditions. The gene Patatin-like protein 2 (PLP2), related to lipid metabolism, was proposed to be targeted by miR6478 (Chen et al., 2020). DNA-directed RNA polymerase II has been reported to be targeted by miR172 in the genus Mimulus (Barozai et al., 2011).

This study provided small RNA libraries available for the scientific community for further analysis of miRNA regulation, at the time that it postulates and validates the presence in the genome of the facultative apomictic cultivar Don Walter of five conserved and two novel miRNAs, given by their evidenced pri-miRNA, pre-miRNA and hairpin structures, being functional as mRNA expression repressors. Moreover, a Kinesin gene expression could be related to miRNA differential regulation in the obligate apomictic genotype Tanganyika.

This work contributed new supporting evidence about the participation of miRNAs in the regulation of genes that are determinant for reproduction in *E. curvula*.

## Data availability statement

The datasets presented in this study can be found in online repositories. The names of the repository/repositories and accession number(s) can be found in the article.

## Author contributions

Conception: IG, VE. Experimental design: IG, JC. Materials: JS, JR, DZ, JC. Experimental procedures: MP, JG, JC. Bioinformatic data processing: JC, IG. Analysis and interpretation: IG, JC, MP. Writing: IG, JC, MP. Critical review: VE. All authors contributed to the article and approved the submitted version.

## Funding

PIP 2021 – 2023 (Code: 11220200100306CO) Identificación de interacciones miRNA-mRNA basada en el análisis del degradoma orientada a la caracterización de su rol en la regulación epigenética de la apomixis diplospórica en pasto llorón (Eragrostis curvula) to IG: laboratory supplies financement. PICT Raíces 2017 - 0879. Genómica estructural para acceder a la región condicionante de la apomixis en Eragrostis curvula. to VE and Mario Cáccamo (NIAB, UK): laboratory supplies and sequencing services financement H2020-MSCA-RISE-2019, Mechanisms of Apomictic Developments (MAD) to VE in Argentina: sequencing services and open access publication fees. PGI 24/A261 Estudio de las bases genéticas y epigenéticas asociadas a la apomixis en Eragrostis curvula (Shrad.) Nees to V.E: laboratory supplies.

## Conflict of interest

The authors declare that the research was conducted in the absence of any commercial or financial relationships that could be construed as a potential conflict of interest.

## Publisher’s note

All claims expressed in this article are solely those of the authors and do not necessarily represent those of their affiliated organizations, or those of the publisher, the editors and the reviewers. Any product that may be evaluated in this article, or claim that may be made by its manufacturer, is not guaranteed or endorsed by the publisher.

## Supplementary material

Supplementary Figure 1 Heatmap of the clustering of samples based on distance matrix. Heatmap showing the distance matrix obtained on logarithm transformed counts, showing similarities and dissimilarities between the seven samples DW-1, DW-2, DW-3, T.USDA-1, T.USDA-2, OTA-1, OTA-2. In the color scale, red and blue represent more and less distance between samples, respectively.

Supplementary Figure 2 Principal component analysis plot. PCA plot showing the similarity within samples with the same reproductive behavior. The variance of principal components PC1 and PC2 are represented in the x and y axis, respectively.

Supplementary Figure 3 Alignment between miRNAs and their targeted transcripts. miRNA - target interaction between. **(A)** Ecu-miRNA_novel_1 and kinesin-like protein KIN-14D, **(B)** Ecu-miRNA_novel_2 and patatin-like protein 2, **(C)** Ecu-miR156c and Growth-regulating factor 8, **(D)** Ecu-miR398 and DREB2A, **(E)** Ecu-miR166 and DNA-directed RNA polymerase II subunit RPB1, **(F)** Ecu-miR2275c and Transcription factor GAMYB, and **(G)** Ecu-miR156d and Squamosa-promoter binding protein-like protein 13 (**Table 4**).
