# Supplementary figures and images for: A combined transcriptome - miRNAome approach revealed that a kinesin gene is differentially targeted by a novel miRNA in an apomictic genotype of Eragrostis curvula
Source: Front Plant Sci. 2022 Sep 30;13:1012682. doi: 10.3389/fpls.2022.1012682 (PMC9563718; doi:10.3389/fpls.2022.1012682)

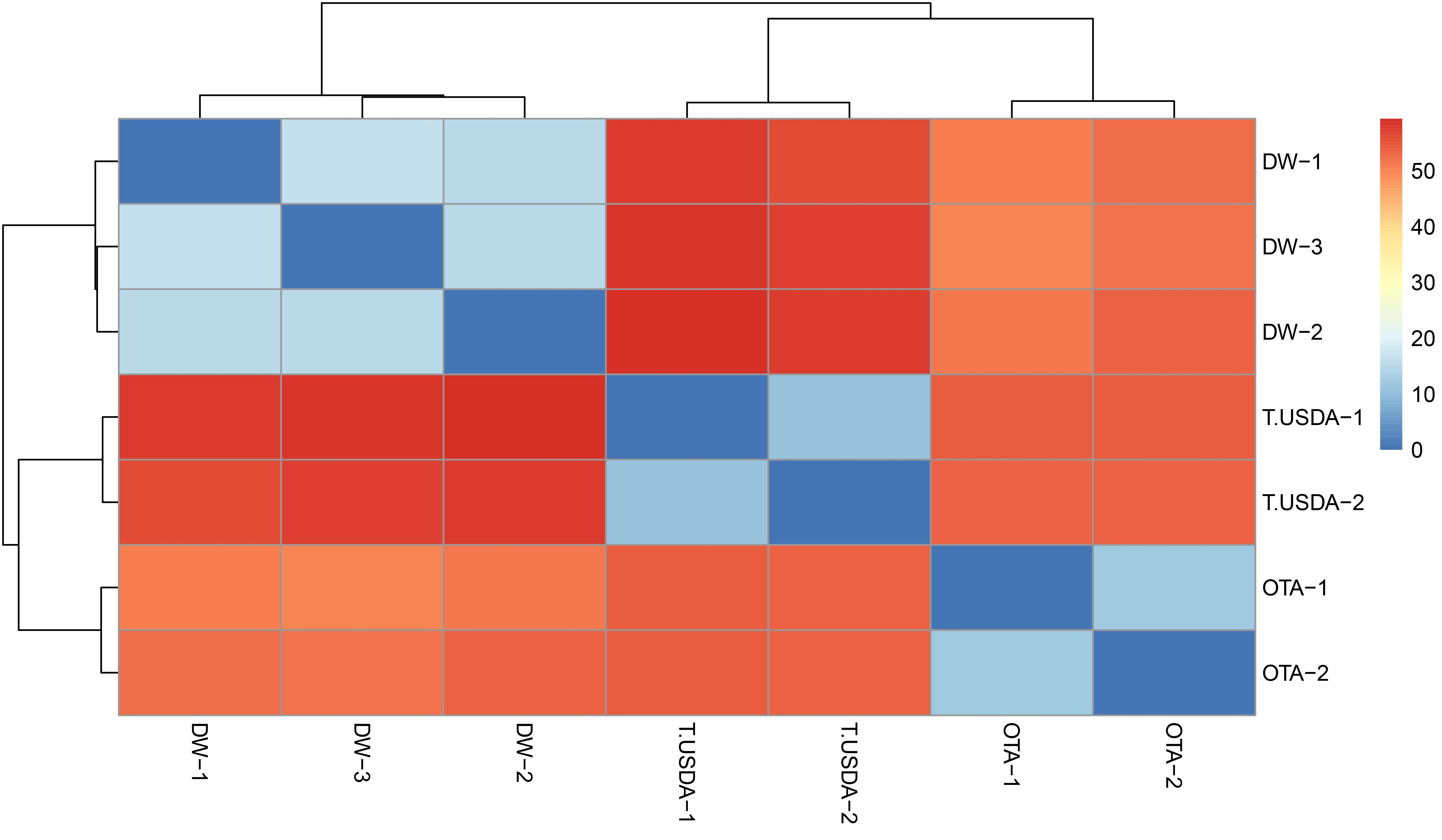

Supplement: Supplementary Figure 1 — Heatmap of the clustering of samples based on distance matrix. Heatmap showing the distance matrix obtained on logarithm transformed counts, showing similarities and dissimilarities between the seven samples DW-1, DW-2, DW-3, T.USDA-1, T.USDA-2, OTA-1, OTA-2. In the color scale, red and blue represent more and less distance between samples, respectively. [file Image_1.jpeg]

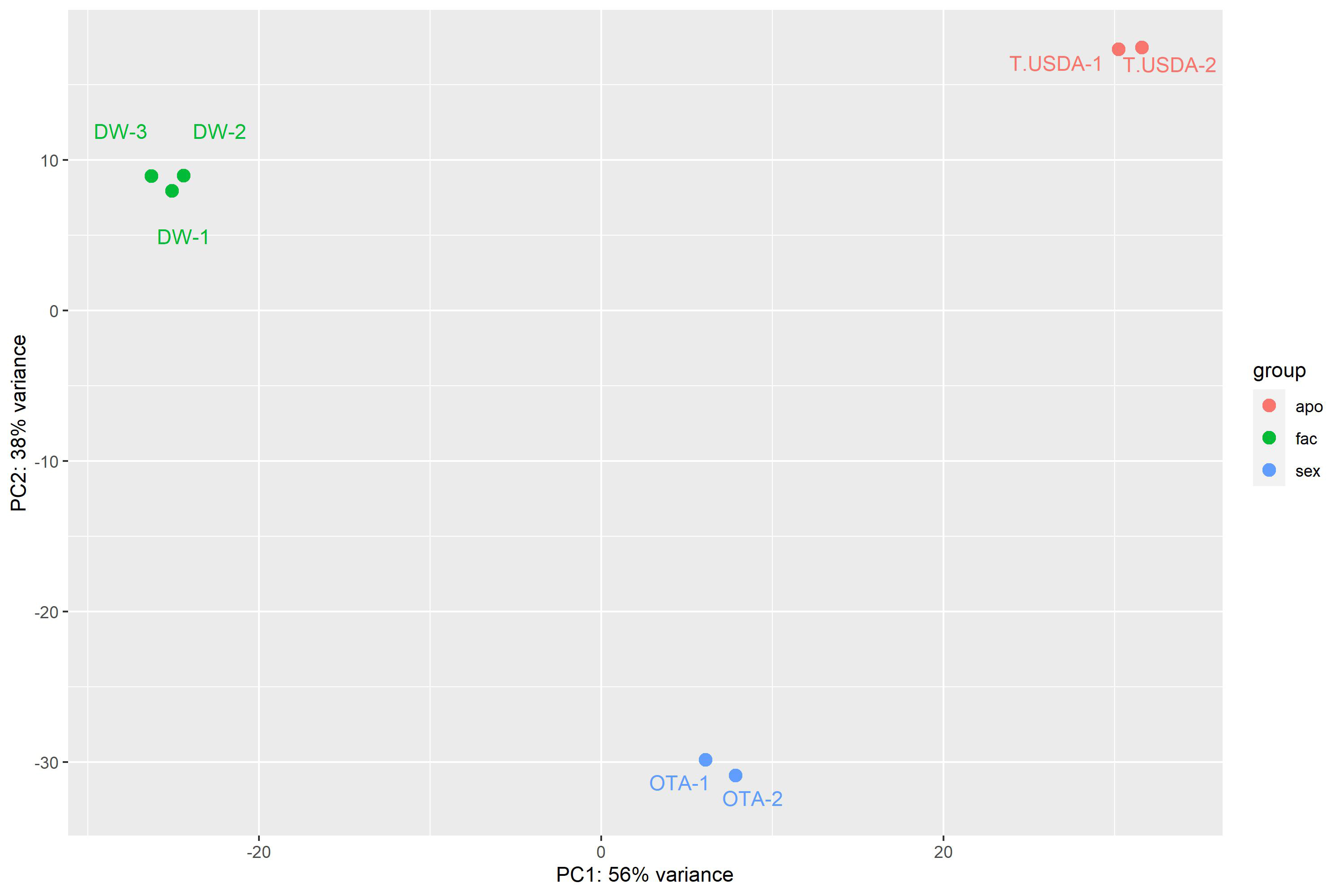

Supplement: Supplementary Figure 2 — Principal component analysis plot. PCA plot showing the similarity within samples with the same reproductive behavior. The variance of principal components PC1 and PC2 are represented in the x and y axis, respectively. [file Image_2.jpeg]

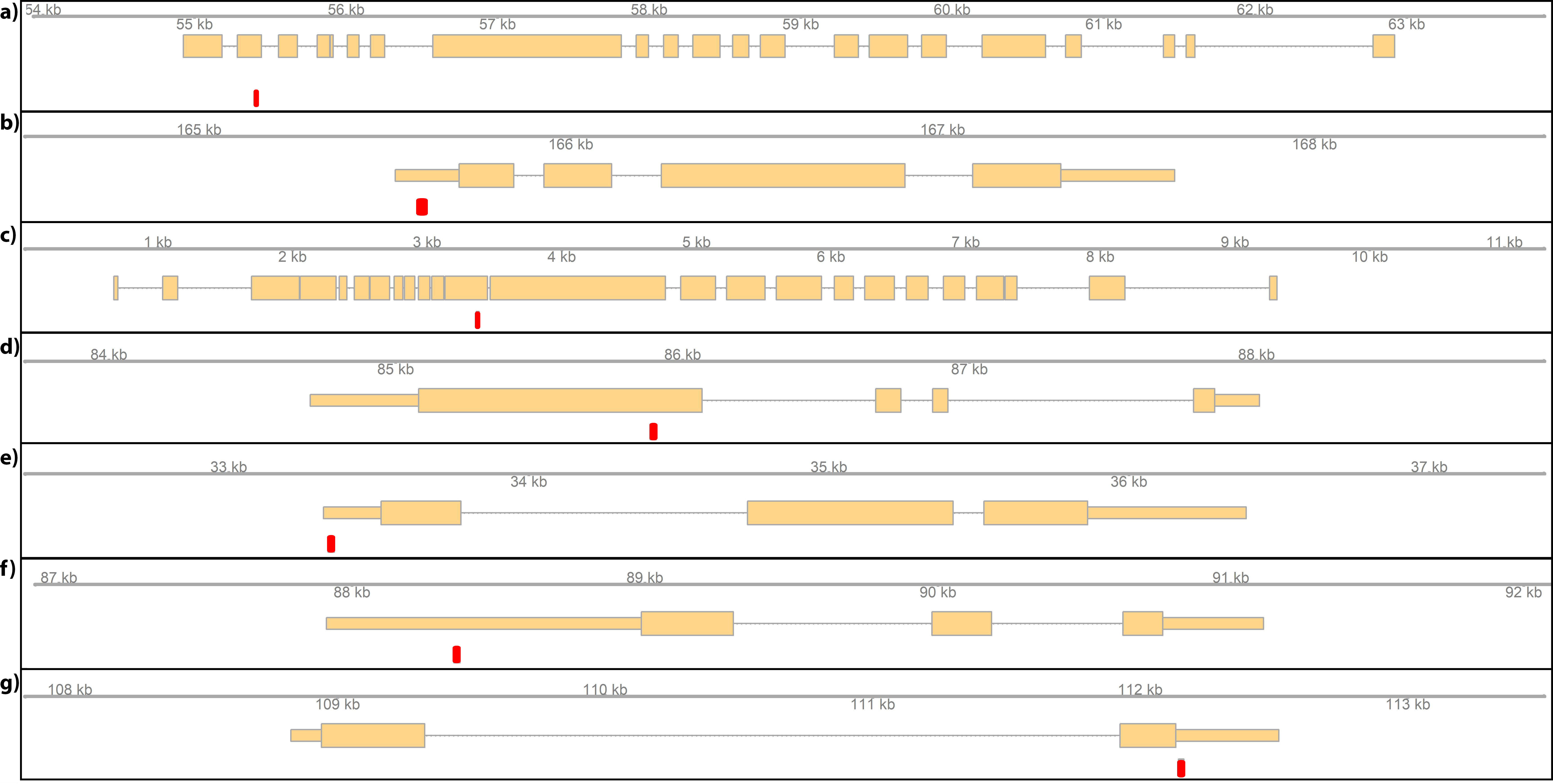

Supplement: Supplementary Figure 3 — Alignment between miRNAs and their targeted transcripts. miRNA - target interaction between. (A) Ecu-miRNA_novel_1 and kinesin-like protein KIN-14D, (B) Ecu-miRNA_novel_2 and patatin-like protein 2, (C) Ecu-miR156c and Growth-regulating factor 8, (D) Ecu-miR398 and DREB2A, (E) Ecu-miR166 and DNA-directed RNA polymerase II subunit RPB1, (F) Ecu-miR2275c and Transcription factor GAMYB, and (G) Ecu-miR156d and Squamosa-promoter binding protein-like protein 13 ( Table 4 ). [file Image_3.jpeg]
